# The Problems of Modern Psychiatry—III

**Published:** 1911-11-25

**Authors:** Thomas Clouston

**Affiliations:** late Physician-Superintendent Royal Asylum, Edinburgh, and Lecturer on Mental Diseases, University of Edinburgh, etc.


### THE PROBLEMS OF MODERN PSYCHIATRY—III.

An Introductory Lecture to the Class of Mental Diseases, University of Edinburgh.

By Sir THOMAS CLOUSTON, M.D., LL.D., late Physician-Superintendent Royal Asylum, Edinburgh, and Lecturer on Mental Diseases, University of Edinburgh, etc.

### DIFFICULTIES OF DIAGNOSIS.

THE physician is sometimes greatly puzzled to distinguish between what may be called true technical insanity and various other states in which the mental action of the brain is disturbed. For instance, in the delirium of fevers, inflammations, gross organic diseases of the brain, cerebral shock from mental or bodily causes, hysteria, religious and other mental excitement and stresses in neurotic people, and traumatisms we may have marked mental symptoms which it would be improper to diagnose or treat as technical insanity, or to think of sending to a mental hospital. I have had cases suffering from all those conditions certified as insane and sent to asylums, and I have sometimes had great difficulty in saving the reputation of my professional brethren from having made incorrect diagnoses.

It is to be remembered, however, that all these conditions may sometimes be the preludes which end in true technical insanity, requiring certification and a mental hospital. The medico-legal question of certification under the Lunacy Acts comes in as a disturbing and very important factor in the consequences of a mistaken diagnosis.

In dealing with persons of a markedly neurotic and unstable brain constitution, and also in dealing with persons of unevolved races, one is apt to meet with transitory conditions which closely resemble true insanity. Certain tribes, such as the Esquimaux and some of the African negroes, will "go mad" from most insufficient causes for short periods and fully resume their normal condition in a few hours.

### DEGENERACY.

There are a whole series of conditions which fall under the term "degeneracy" to which attention has been markedly drawn of late years as one of the problems of psychiatry. We call members of this class "degenerates." They all take their origin in hereditary defects, and mostly show some signs of the condition from an early period of life.

The conditions are incapable of definition; they are so wide and far-reaching. In most of them there are bodily departures from the ideal and normal, which we call "stigmata of degeneration." Faults in the shape or size of the head, in the special sense organs, in the face, in the mode of action of the "mind muscles," in attitudes and movements, may all constitute those stigmata. Faults of mental reaction, moral conduct, social instincts and conduct are the chief ways in which degeneracy shows itself.

### BACKWARD EVOLUTION-REVERSION.

There is one consideration which it is well to remember in considering the causation of mental disease. The brain cell being the highest organic structure in nature, in the course of its evolution it has had to undergo, to reach its present stage, the severest tests of natural selection during the ages that have elapsed since nerve tissue first appeared in animals. It has had to make adaptations and re-adaptations to innumerable different environments.

### A PHYSIOLOGICAL LAW.

Now it may be considered a physiological law that the greater the integration of any form of cell or structure, the greater the risk there is of ontogenetic and phylogenetic degeneration. The brain cell has gradually acquired a sensitiveness to outside stimuli and outside agents which no other cell possesses. It is so highly organised that it is subject to the most delicate disturbing agencies. Its mental functions alone render it subject to influences to which no other cell responds. During those processes of *cell evolution* and *adaptation to environment*, it is subject from that cause to failures and liabilities, to disturbances and to destruction of its mental function.

Speaking generally, I consider that many cases of St. Vitus' dance, much of the epilepsy, some of the adolescent insanities, and most of the cases of dementia præcox, are the result primarily of the *incapacity* of the brain cell *to adapt itself to its environment*. It is a well-known fact that though mental disease is more uncommon in the primitive and savage peoples than it is in the highly civilised nations, yet when the primitive man is suddenly transferred into the life of the civilised city and has to take part in its social life, there is considerable danger of mental upset.

### STAGES OF MENTAL DISEASE.

In psychiatry it is always most important to realise that most cases pass through various stages—the prodrometal, the initial, the fully developed, and the convalescent. The prodrometal stage is the most important on the whole for curative treatment.

The prodromata of mental disease usually consist of sensory symptoms, such as headache, neuralgia, and paræsthesia in various organs; of nutritive symptoms, such as falling-off in flesh, and neurasthenia; of blood changes, such as anæmia and leucocytosis. Especially the symptom of anorexia is extremely common. Then there are minor mental symptoms which are usually not counted as disease at all, such as deadness of mental feeling, over-sensitiveness, irritability.

### MUSCULAR EXPRESSIONS OF MIND.

All normal mind exhibits itself in a normal expression of face and eye and in natural postures and gestures. It is a most important thing in psychiatry to observe those muscular expressions of mind and to estimate their signification. Among the innumerable debts that psychology owes to Darwin, his work on the "Expression of the Emotions" is one of great importance. There are in the human subject between fifty and sixty muscles, many of them of extreme tenuity, the function of which is chiefly to express mind. I am in the habit of calling them "mind-muscles." They have an enormous supply of motor innervation for their size, the motor strands being connected directly with the mental cortex. They have the power of expressing almost every shade of depression, exaltation, of anger, and lethargy, of suspicion, and confidence which the human being can feel.

In most forms of insanity their action is changed. The eye expression is a most subtle and most important symptom, the precise mechanism of which we have been yet unable fully to elucidate.

### Different Standards of Mind.

Speaking generally, the whole class of patients suffering from depressive states of mind are not regarded as insane till they become strongly suicidal or excited in conduct. It is this misapprehension which is the cause of vast numbers of suicides. The doctor is not consulted nor precautions taken until it is too late.

### Ideas of what a Mental Hospital Is.

The most mistaken and preposterous ideas prevail among a large section of the public as to what a modern mental hospital is. The old ideas of "madness" and a "madhouse" are still prevalent at the present day. I need hardly say to you that a mental hospital is an institution specially adapted for treating the special disease of insanity, and differs in no essential respects from a general hospital or a special hospital for fevers, etc. You will have this abundantly confirmed in your visits to Morningside.

It is our duty as medical men to take special pains to disabuse the public of their wrong ideas on this point, because they cause infinite and unnecessary pain to the relatives of patients, and to the patients themselves, and they prevent early treatment, which means effectual treatment, in those hospitals at the most curable stage in the disease. If mental hospitals were regarded in the same way as ordinary and special hospitals, an enormous boon would be conferred on suffering humanity.

### Ætiology.

To enable you to form a general idea of the causation of mental disease I shall take two or three typical examples. In the first place it is necessary to assume that in by far the greater number of cases of typical mental disease the cellular element, which is the vehicle of mind, has through a bad heredity acquired the inherent quality of a morbid instability.

This means that it is specially weak in its resistive power to the action of toxines, to unusual stimuli, and to mental stress. Its defences are weak. To a brain so constituted let us suppose that alcohol is introduced through the blood in excessive quantity and more or less continually. Each brain undoubtedly possesses its special resistive power in regard to toxines. In one man the mind cells select and attract more than their share of the alcohol circulated in the blood, and you have resulting stupidity or mental exhilaration, or delirium, while the individual is able to walk and write and talk fairly well. In another case the motor centres may select and attract more than their share, and the man remains fairly intelligent, but cannot walk or write. The former case is the one that best illustrates the action of a toxin in producing subsequent insanity.

### Toxic Effects.

The molecules of the toxin come into the most intimate relationship with the excessively mobile molecules of the brain cell and the effects are an undue stimulation, irritation, and dilatation of the capillaries, diminished peri-vascular spaces, and marked hypertrophy of the neuroglia. In short, the man becomes insane, and we call it "alcoholic insanity."

In most such cases of alcoholic insanity there are hallucinations of hearing—that is to say, the man continually imagines he hears people speaking to him when no such thing exists. What has happened to produce this particular mental symptom? The alcohol has produced a special irritation in the auditory centres of the cortex, so that they act without the stimuli of sounds from without. His brain, as it were, speaks to his mind, and his mind cells, having undergone disease, are not able to exercise judgment and perception, so that the man believes in the real existence of those subjective sensations of his.

*Delusion.*—One form of insane delusion consists of a misinterpretation by the mind of real sensations in the body. For instance, a patient of mine had the delusion that the devil was inside her. She had undoubtedly real pain in the gastric region, and after a time we discovered that she had a cancerous growth in the pylorus. Another case believed that she was eaten up by serpents in the region of her heart; as a matter of fact, she had angina pectoris.

There is no more important thing to be kept in mind in examining our delusional cases than to find out if there is such a bodily cause for such delusion, and the action of various toxins which cause such bodily pains or discomforts has always to be kept in mind in delusional or hallucinatory cases.

### Urgent Problems.

The urgent problems of modern psychiatry at present are psycho-physiological and bio-chemical, and not histological. We cannot explain mental and living processes by the examination of dead structures. In this consists the importance of the experimental work in brain toxins of Kraepelin and MacDougall; of the chemical work of most of the elaborate "tests" of brain conditions by Wassermann and others, and of the bacteriological and the anti-toxin and serum experimental work of Ford Robertson in general paralysis. In this lies the value of this modern clinical methods where the observations at the bedside are combined with those of the laboratory. We have to solve the questions of why this human brain cortex is so liable to the special disorder of action we call insanity. Why the human mind is thereby so affected that man loses the highest qualities that evolution has brought him, and how this terrible result can be prevented or remedied.

